# Association between coverage of maternal and child health interventions, and under-5 mortality: a repeated cross-sectional analysis of 35 sub-Saharan African countries

**DOI:** 10.3402/gha.v7.24765

**Published:** 2014-09-03

**Authors:** Daniel J. Corsi, S. V. Subramanian

**Affiliations:** 1 Center for Population & Development Studies, Harvard School of Public Health, Cambridge, MA, USA; 2 Department of Social and Behavioral Sciences, Harvard School of Public Health, Boston, MA, USA

**Keywords:** Africa, child mortality, child health, maternal and child health interventions, low-income countries, trends, socioeconomic factors

## Abstract

**Background:**

Infant and child mortality rates are among the most important indicators of child health, nutrition, implementation of key survival interventions, and the overall social and economic development of a population. In this paper, we investigate the role of coverage of maternal and child health (MNCH) interventions in contributing to declines in child mortality in sub-Saharan Africa.

**Design:**

Data are from 81 Demographic and Health Surveys from 35 sub-Saharan African countries. Using ecological time-series and child-level regression models, we estimated the effect of MNCH interventions (summarized by the percent composite coverage index, or CCI) on child mortality with in the first 5 years of life net of temporal trends and covariates at the household, maternal, and child levels.

**Results:**

At the ecologic level, a unit increase in standardized CCI was associated with a reduction in under-5 child mortality rate (U5MR) of 29.0 per 1,000 (95% CI: −43.2, −14.7) after adjustment for survey period effects and country-level per capita gross domestic product (pcGDP). At the child level, a unit increase in standardized CCI was associated with an odds ratio of 0.86 for child mortality (95% CI: 0.82–0.90) after adjustment for survey period effect, country-level pcGDP, and a set of household-, maternal-, and child-level covariates.

**Conclusions:**

MNCH interventions are important in reducing U5MR, while the effects of economic growth in sub-Saharan Africa remain weak and inconsistent. Improved coverage of proven life-saving interventions will likely contribute to further reductions in U5MR in sub-Saharan Africa.

Infant and child mortality rates are among the most important indicators of child health, nutrition, implementation of key survival interventions, and the overall social and economic development of a population (1). In September 2000, the governments of 147 countries agreed to accelerate efforts to achieve a series of development goals (2), now referred to as the Millennium Development Goals (MDG) (3). Of the eight goals established, the fourth (MDG-4) was ‘to reduce by two thirds, between 1990 and 2015, the under-five mortality rate’ (U5MR) (3). Considerable resources and efforts have gone into assessing the progress toward achieving MDG-4, including determining what interventions are needed to accomplish this goal (4, 5). With less than 1,000 days remaining until the 2015 deadline for accomplishing the goals of MDG-4, there has been renewed attention to the success (or not) of meeting these targets (6).

According to the most recent estimates from the UN Inter-agency Group on Child Mortality Estimation (UN-IGME), the region of sub-Saharan Africa had the highest rate of child mortality (121 deaths per 1,000 live births) in 2010 (7), equivalent to one child in eight children dying before their fifth birthday. This rate was nearly double the average in developing/low-income regions (62.7 deaths per 1,000 live births), and nearly 18 times the average for developed/high-income regions (6.8 per 1,000). Overall in sub-Saharan African countries, the U5MR declined from 174 per 1,000 in 1990 to 121 per 1,000 in 2010 (7). This corresponds to a 30% reduction in U5MR since 1990, well short of the MDG target of a two-thirds reduction (4). The annual rate of reduction in U5MR has been 1.8% over the period 1990–2010, although this increased to 2.4% during the decade from 2000 to 2010. Compared with other developing regions, however, sub-Saharan Africa has experienced slower rates of decline in U5MR and continues to have higher fertility rates.

A policy alternative to economic growth (8–10) for improvements in health is to encourage strengthening of health systems in low-income countries (11), and in particular improving the coverage of key maternal, newborn, and child health interventions (12, 13). Coverage of interventions such as measles and diphtheria, pertussis, and tetanus (DPT) vaccinations, and skilled birth attendance are widely accepted indicators of progress toward improving health systems and achieving the MDG (14, 15). Further, the coverage and equity distribution of a set of core maternal and child health (MNCH) interventions are being tracked across a range of low- and middle-income countries as we approach the 2015 MDG deadline (16). To date, however, there has been limited evidence on how the coverage of these interventions is related to declines in U5MR, as these measures are considered long-term indicators of health systems performance and are insensitive to shorter-term changes in coverage (17).

In this paper, we investigate factors that have contributed to the recent declines in U5MR in sub-Saharan Africa since 1990, using data from the Demographic and Health Surveys (DHS). Specifically, we focus on the contributions of MNCH interventions in reducing child mortality in sub-Saharan Africa within a hierarchical framework where country-level factors relating to economic growth, and coverage of MNCH interventions are treated as ‘distal’ determinants for child mortality (Fig. 1). We examined a set of eight MNCH interventions that can be estimated from the DHS data and summarized in a composite coverage index (CCI) (12): family planning needs satisfied (FPS); antenatal care with a skilled provider; skilled birth attendance; DPT, measles, and BCG (tuberculosis) vaccination; oral rehydration therapy (ORT) for children with diarrhea; and care seeking for pneumonia (CPNM) (21–37).

**Fig. 1:**
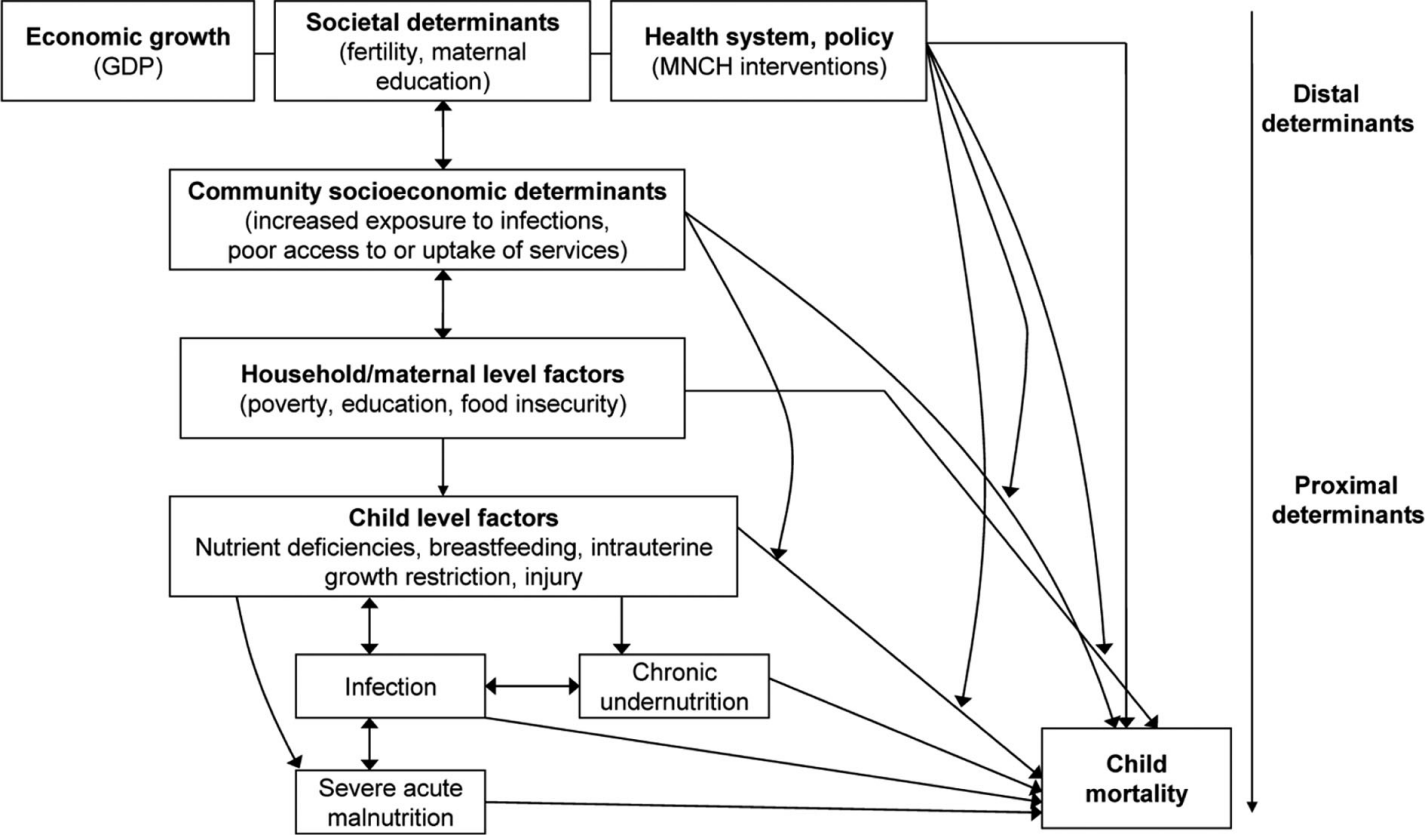
Hypothesized relational structure between child mortality; child-, maternal-, household-, and community determinants and country-level economic growth; social determinants; and health system and policy variables. Country-level factors may: (1) exert direct, cross-level effects on child mortality, (2) exert indirect, cross-level effects mediated through more proximal variables, such as community or household socioeconomic factors, and (3) modify the associations between independent and dependent variables operating within or across levels (e.g. country gross domestic product (GDP) modifying the effects of household-level disadvantage on child mortality). This conceptualization is complex and many interactions may produce bidirectional effects such that family-level and child-level factors may influence the community- and country-level variables. [Adapted from Bronfenbrenner (18), Mosley & Chen (19), Boyle et al. (20), and Bhutta et al. (21)].

## Methods

### Data sources

We extracted data from DHS surveys (38) conducted since 1990. DHS are household surveys that use nationally representative sampling plans and have special emphasis on fertility, child mortality, and indicators of MNCH (38). We selected standard surveys for each country that included birth histories (‘BR’ files from which child mortality rates could be calculated) of women aged 15–49 and MNCH coverage indicators. In total, 81 surveys were included, covering 35 countries and 93% of the population of sub-Saharan Africa (39). Twenty-four surveys were conducted between 1992 and 2000, 20 between 2000 and 2004, 21 between 2005 and 2008, and 16 since 2009. Twenty-four of the 35 countries conducted at least two surveys during this period, and 22 conducted three or more.

### Study population, data designs, and sample sizes

The study population was structured as two distinct data designs. First, we examined the study population as an ecological time-series design with countries repeatedly observed over time. In this design, the lowest level of analysis was the survey period, nested within countries as a hierarchical structure. Second, we used a repeated cross-sectional design, with children at the lowest unit of analysis. A key substantive advantage of the second approach is the ability to account for within-country between-child factors that can influence both child mortality and the country-level economic development and coverage indicators. Further, the ecological time-series data structure assumes that the probability of dying (or U5MR) is the same for all children within a country period. This assumption is relaxed in the second data structure, although in doing so we are modeling the probability of a child dying before the fifth birthday, and not U5MR.

In the ecological time-series design, 81 survey periods were available for analysis, covering 35 countries, with an average of 2.3 surveys per country. For the child-level analyses, children across all surveys were pooled, and the probability of child death was examined in the 3-year period immediately preceding the survey. In total, there was information on 395,493 children born within the reference period. After making exclusions for missing data on covariates, the final analytical sample size was 393,934.

### Outcomes

This study uses two outcomes, corresponding to the two data designs employed. In the ecologic time-series design, the outcome is U5MR for the 3 years reference period in each survey. In the child-level design, the outcome is the probability of child death occurring within 3 years prior to the survey. At an aggregate level, child mortality is typically expressed as probabilities of dying between exact ages (*x* and *x*+*n*), which are derived from life tables and denoted by _*n*_ *q*_*x*_ (40). The U5MR, also denoted _5_ *q*_0_, is formally defined as the probability a child death occurring between birth and a child's fifth birthday, expressed as deaths per 1,000 live births (7, 40). U5MR is a composite measure of mortality occurring during the first 5 years, which can be further defined as the probability of dying within 1 month (neonatal mortality), 0–11 months (infant mortality, including neonatal deaths, or _1_ *q*_0_), and 12–59 months (child mortality, conditional on having reached the first birthday, or _4_ *q*_1_) (41).

U5MRs were calculated using the DHS synthetic cohort life table methodology (42). This approach uses age segments 0, 1–2, 3–5, 6–11, 12–23, 24–35, 36–47 months (completed ages) for the calculation of the individual probabilities of dying, without adjustment for age of death heaping at 12 months. Such heaping may occur during fieldwork when deaths occurring slightly prior to or after 12 months of age are reported as a 1 year age of death (42). Therefore, some deaths that are actually infant deaths are shifted up to age 1. The analyses in this paper, however, were on all under 5 deaths, and any heaping would have little influence on the results. Imputation procedures were used for children with missing ages at death. On average, only small numbers of children in the DHS (about 1 in 1,000) reported to have died were not given an age at death and had the age of death imputed (T Pullum, year of personal communication was 2014). The imputation procedure involved finding a range of dates within which death could have occurred, and then selecting a value randomly within that range which would likely not introduce any upwards or downwards bias. The calculation of the U5MR was based on the number of deaths to live-born children in a 3-year reference period preceding the survey. Death probabilities were calculated for each of the age segments defined above and then combined into the mortality rate as the product of the component survival probabilities, and expressed as a rate per 1,000 live births.

In the child-level design, the outcome was defined as a child death occurring within the reference period. This was expressed as a binary outcome: 1 for a death occurring in the child's first 5 years; 0 for survival through 37 months of age.

### Exposure

Our key exposure of interest was coverage of MNCH interventions. Based on prior literature, we selected eight established interventions that have sufficient evidence of an effect on reducing child mortality from the major causes of under-5 deaths and can be summarized as a composite index for comparability between countries and within countries over time (12, 22–37, 43). The interventions included were: FPS, skilled birth attendant at delivery (SBA), at least one antenatal care visit with a skilled provider (ANCS), three doses of diphtheria-pertussis-tetanus (DPT3) vaccine, measles vaccination (MSL), BCG (tuberculosis) vaccination (BCG), ORT for children with diarrhea, and CPNM. The coverage of these interventions at a country level was summarized using the CCI, which is based on the following weighed average of the eight interventions (12):

1 $$\text{CCI}=\frac{1}{4}\left(\text{FPS}+\frac{\text{SBA}+\text{ANCS}}{2}+\frac{2\text{DPT}3+\text{MSL}+\text{BCG}}{4}+\frac{\text{ORT}+\text{CPNM}}{2}\right)$$

The CCI gives equal weight to family planning, maternal and newborn care, immunization, and case management of sick children, and has been proposed as an effective way summarize and compare coverage of MNCH interventions across countries and over time (12).

### Covariates

At the country level, per capita gross domestic product (pcGDP) was used as the primary measure of a country's economic growth and development. These data were obtained from the Penn World Tables (44) and were lagged 2 years from the date at which the survey began. Analysis of pcGDP was included in regression models as the logarithm (base 10) of pcGDP. At the child level, we used a variety of theoretically important maternal and child characteristics as covariates (45). Age, sex, multiple/single birth, birth order, and preceding birth interval were included as child characteristics; age of the mother at birth, maternal education, household wealth quintile, area of residence were included as maternal/household-level characteristics. Household wealth was defined according to an index developed from indicators of household asset ownership and housing characteristics (e.g. whether the household had a flush toilet, refrigerator, car, moped/motorcycle, television, washing machine, or telephone). Country-specific and weighted linear combinations of these items were constructed with weights for each item obtained from a principal component analysis (46). The index was then standardized, and using the quintiles of this distribution, the survey population in each country was divided into fifths from poorest to richest. Similar measures have been developed in India and other settings and have been shown to be a consistent proxy for household income and expenditure (47).

### Statistical analyses

We conducted two separate set of analyses corresponding to the two data structures described previously. For the ecological time-series data, we fit linear regression models of the form:

2 $${\text{y}}_{\text{ij}}=\beta_{\text{0}}+\text{B}{\text{C}}_{\text{j}}+\text{B}{\text{S}}_{\text{ij}}+\beta_{\text{1}}\text{CC}{\text{I}}_{\text{ij}}+{\text{e}}_{\text{0ij}}$$

where y_ij_ represents the U5MR for survey time i in country j; β_0_ represents the constant or the average U5MR holding CCI constant, and after accounting for country differences (BC_j_);BC_j_ represents the country-specific dummy variables estimating differences in U5MR between countries; BS_ij_ represents the effects associated with dummies for survey years; β_1_CCI_ij_ represents the change in U5MR for a unit change in CCI; and e_0ij_ represents the residuals at the survey-year level i in country j.

A second series of analyses were conducted child-level dataset. In these analyses, the basic model is a logistic regression model with a binary response (*y=*1 for child death during the reference period, *y=*0 otherwise). Countries are treated as fixed effects using country indicator variables in the fixed part of the model (BC_j_). The outcome of child mortality, Pr(*y*_*ij*_=1), is assumed to be binomially distributed y_ij_~Binomial (1,π_ij_) with probability π_ij_ related to the set of independent variables X and a random effect for each level by a logit link function:

3 $$\text{Logit}(\pi_{\text{ij}})=\beta_{\text{0}}+\text{B}{\text{C}}_{\text{j}}+\text{B}{\text{S}}_{\text{ij}}+\beta_{\text{1}}\text{CC}{\text{I}}_{\text{ij}}+\text{B}{\text{X}}_{\text{ij}}$$

The intercept, β_0_, represents the log odds of child mortality for the reference group, BS_ij_ is a vector of coefficients for dummy variables for survey years, β_1_CCI_ij_ represents the log odds of child mortality for a one-unit increase in CCI, and the BX represents a vector of coefficients for the log odds of child mortality for a one-unit increase for each independent variable. Models were weighted and standard errors adjusted for the complex multistage sampling design of the surveys. Coefficients were exponentiated and presented as odds ratios with 95% confidence intervals.

## Results

Between 1992 and 2012, the U5MR declined in a majority (19 of 24) of sub-Saharan African countries where repeated DHS surveys were available, although the rate of change varied across countries (Table 1). The U5MR varied between 62.8 deaths per 1,000 live births in Sao Tome to 305.8 per 1,000 in Niger in the initial round of surveys (median year: 1998), corresponding to a five-fold difference across countries. In the most recent round of DHS surveys (median year: 2005), the U5MR ranged from 67.2 deaths per 1,000 live births in Senegal to 190.1 per 1,000 in Chad, indicating a three-fold difference across countries. During this period, the CCI increased in 17 countries from an average of 53.4% (SD 14.4) among all countries in the first survey period to 58.7% (SD 12.2) among the most recent wave in 24 countries.

**Table 1:** Country, year of survey, sample size, % under-5 child deaths, U5MR, CCI, and log per capita GDP in 35 sub-Saharan African countries for the first and most recent survey during the period 1990–2012

	Baseline survey						Repeated survey					
	
Country	Year	*N*	% Child deaths	U5MR	CCI	log pcGDP	Year	*N*	% Child deaths	U5MR	CCI	log pcGDP
São Tomé and Príncipe	2008	1,238	3.9	62.8	74.7	7.3	–	–	–	–	–	–
Namibia	2000	2,570	4.5	66.6	69.4	8.2	2006	3,336	5.3	69.3	76.7	8.4
South Africa	1998	3,237	5.3	68.1	77.1	8.6	–	–	–	–	–	–
Zimbabwe	1994	2,537	6.1	82.6	73.4	6.2	2010	3,723	6.8	91.9	71.1	5.6
Congo	2012	9,180	5.8	85.0	67.5	7.7	–	–	–	–	–	–
Gabon	2000	2,045	7.1	91.3	62.4	9.4	2012	3,988	5.2	72.1	62.4	9.2
Burundi	2010	4,932	6.4	92.7	67.3	6.0	–	–	–	–	–	–
Liberia	2007	3,592	7.0	99.8	51.0	5.9	–	–	–	–	–	–
Comoros	1996	1,205	7.9	100.0	53.7	7.0	–	–	–	–	–	–
Ghana	1998	2,046	7.1	106.9	52.0	7.2	2008	1,871	5.4	77.8	56.6	7.4
Kenya	1998	3,612	7.5	110.5	66.3	7.0	2008	3,844	6.0	77.5	67.4	7.1
Senegal	2005	7,140	6.5	113.0	55.7	7.2	2010	7,888	4.6	67.2	60.4	7.3
Lesotho	2004	2,397	8.5	115.5	67.6	7.1	–	–	–	–	–	–
Swaziland	2006	1,824	9.5	127.0	75.3	8.0	–	–	–	–	–	–
Sierra Leone	2008	3,721	8.6	134.0	51.2	6.7	–	–	–	–	–	–
Tanzania	1996	4,346	9.2	137.7	58.9	6.5	2010	5,018	6.0	84.5	68.6	7.0
Togo	1998	4,303	7.2	137.9	46.2	6.7	–	–	–	–	–	–
Côte d'Ivoire	1994	4,104	8.8	140.1	45.3	7.3	2011	5,059	7.0	102.9	55.5	7.1
Congo DR	2007	5,775	9.9	141.6	53.5	5.3	–	–	–	–	–	–
Cent African Rep	1994	2,909	9.4	143.4	48.9	6.4	–	–	–	–	–	–
Uganda	1995	4,607	9.1	145.4	51.7	6.4	2011	4,941	6.0	93.0	65.9	7.0
Nigeria	1999	3,747	9.7	147.3	44.3	7.0	2008	18,124	9.8	157.2	41.5	7.4
Cameroon	1998	2,414	8.7	149.3	52.5	7.3	2011	7,527	7.1	119.9	59.2	7.5
Madagascar	1997	3,814	9.4	151.0	46.3	6.7	2008	7,736	4.9	69.4	63.2	6.6
Benin	1996	3,130	9.1	152.9	52.3	6.9	2011	8,221	4.7	72.8	56.5	7.1
Ethiopia	2000	6,641	10.7	161.6	22.5	6.0	2011	7,050	6.5	91.0	38.8	6.5
Mozambique	1997	4,294	9.9	168.9	44.4	5.8	2011	7,196	6.4	95.8	59.6	6.6
Burkina Faso	1993	3,533	10.5	179.3	44.1	6.5	2010	9,441	7.3	123.4	64.9	6.8
Guinea	1999	3,659	12.5	181.7	43.0	6.6	2012	4,440	7.1	118.7	44.4	6.7
Malawi	2000	7,835	11.8	186.1	62.0	6.2	2004	7,050	7.4	121.3	64.5	6.1
Chad	1996	4,621	11.8	191.8	23.6	6.7	2004	3,472	12.3	190.1	27.2	6.7
Zambia	1996	4,680	12.6	195.4	67.4	6.6	2007	4,134	7.6	117.1	61.8	7.0
Rwanda	2000	4,870	11.8	198.4	48.3	6.4	2010	5,417	5.1	69.5	73.5	6.9
Mali	1995	6,281	13.5	231.0	34.1	6.6	2006	9,023	10.6	181.5	40.4	6.7
Niger	1992	4,318	16.3	305.8	20.5	6.3	2012	8,037	6.3	124.8	56.4	6.3

Source: Authors’ calculations from DHS data; percent child deaths weighted and calculated in a 3-year window preceding the start of the survey.

U5MR=under-5 mortality rate; CCI=composite coverage index; log pcGDP=logarithm of per capita gross domestic product.

**Fig. 2:**
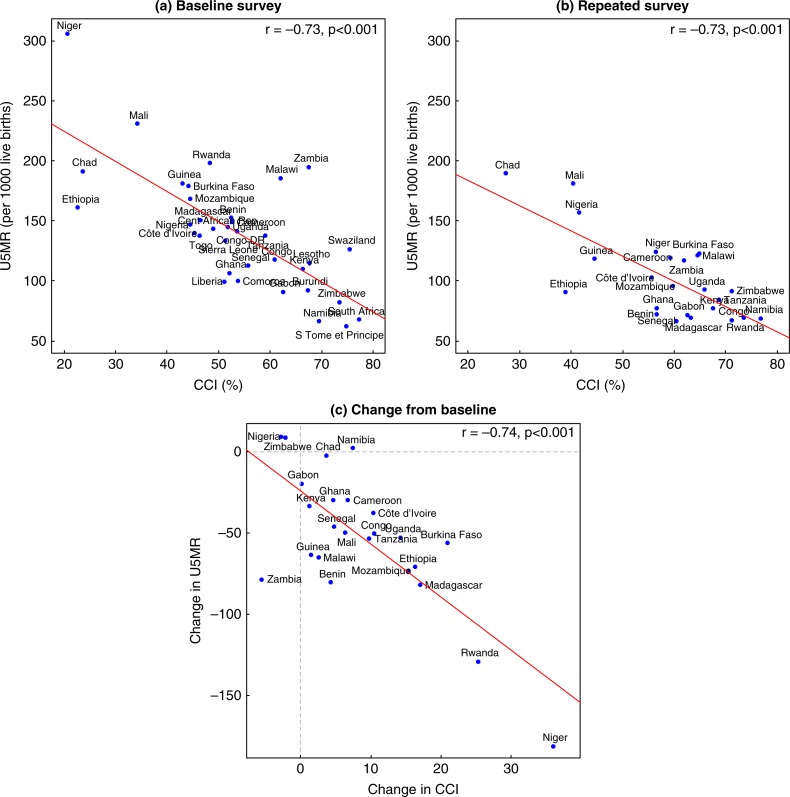
Correlation between under-5 mortality rate (U5MR) and composite coverage index (CCI) at baseline (panel a, *n*=35 surveys) and repeated surveys (panel b, *n*=24) and correlation between the change in U5MR and change in CCI from baseline (panel c, *n*=24).

At both the baseline and repeated surveys, an inverse association was seen between country-level U5MR and CCI coverage, indicating lower rates of under-5 mortality in countries with greater coverage of intervention (Pearson correlation −0.73 at both times, *p*<0.001, Fig. 2a and b). This association held when examining the average changes in U5MR and CCI over time in a subset of 24 countries with repeated surveys (Pearson correlation −0.74, *p*<0.001, Fig. 2c).

At an ecologic level, the regression analyses described in Equation 2 show that a standardized unit increase in CCI was associated with a reduction of 28.5 per 1,000 in U5MR (95% CI: −42.4, −14.6), after accounting for secular declines in U5MR as captured by survey period fixed effects (Table 2). The inclusion of log pcGDP to Model 2 did not substantial alter this effect (β=−29.0, 95% CI: −43.2, −14.7).

**Table 2:** Risks, bivariate odds ratios (OR), and multivariable adjusted odds ratios (aOR) of child mortality according to child-, maternal-, and household-level covariates across 35 sub-Saharan African countries, 1992–2012

Maternal/child covariates	Children, *n*	%	Deaths, *n*	% Risk	OR	95% CI	aOR	95% CI
Total	393,934	100.0	32,747	8.5	–		–	
Survey period								
Baseline (reference)	135,141	34.4	12,639	9.5	1.00		1.00	
1st repeated survey	111,227	28.1	9,614	8.9	0.92	(0.89–0.96)	0.91	(0.87–0.94)
2nd repeated survey	121,349	30.7	9,032	7.6	0.78	(0.75–0.81)	0.75	(0.72–0.78)
3rd repeated survey	26,217	6.8	1,462	5.7	0.57	(0.53–0.61)	0.58	(0.54–0.62)
Sex of the child								
Male (reference)	199,452	50.7	17,629	9.0	1.00		1.00	
Female	194,482	49.3	15,118	7.9	0.87	(0.85–0.90)	0.87	(0.85–0.89)
Multiple birth								
Singleton (reference)	380,785	96.7	29,526	7.9	1.00		1.00	
Multiple birth	13,149	3.3	3,221	25.0	3.88	(3.66–4.10)	3.73	(3.51–3.95)
Birth order								
1st (reference)	83,088	20.9	7,746	9.5	1.00		1.00	
2nd	71,833	18.2	5,295	7.7	0.79	(0.76–0.82)	1.24	(1.18–1.30)
3rd	59,702	15.3	4,426	7.4	0.77	(0.73–0.80)	1.18	(1.12–1.26)
4th	48,989	12.4	3,682	7.6	0.79	(0.75–0.83)	1.18	(1.10–1.26)
5th and higher	130,322	33.2	11,598	9.1	0.95	(0.92–0.99)	1.24	(1.16–1.32)
Birth								
< 24 mo. (reference)	137,523	34.8	14,561	10.8	1.00		1.00	
24–47 mo.	184,609	47.1	13,642	7.5	0.67	(0.65–0.69)	0.58	(0.56–0.60)
48+ mo.	71,802	18.1	4,544	6.5	0.57	(0.55–0.60)	0.52	(0.50–0.55)
Child age								
0–1 year (reference)	249,701	63.5	17,431	7.1	1.00		1.00	
2–3 years	144,233	36.5	15,316	10.8	1.59	(1.55–1.63)	1.35	(1.31–1.39)
Maternal age at child birth								
< 17 years	30,267	7.6	3,369	11.6	1.60	(1.52–1.69)	1.30	(1.21–1.39)
17–19 years	59,366	15.0	5,351	9.2	1.24	(1.18–1.30)	1.10	(1.04–1.17)
20–24 years	108,266	27.6	8,389	7.9	1.05	(1.01–1.09)	1.02	(0.97–1.06)
25–29 years (reference)	90,207	22.9	6,680	7.5	1.00		1.00	
30–49 years	105,828	26.9	8,958	8.6	1.16	(1.11–1.21)	1.15	(1.10–1.20)
Maternal education								
No education (reference)	189,605	48.6	17,746	9.6	1.00		1.00	
Any primary	137,165	34.8	11,165	8.2	0.84	(0.82–0.87)	0.99	(0.95–1.02)
Incomplete secondary	52,894	12.9	3,141	6.0	0.60	(0.57–0.63)	0.76	(0.72–0.80)
Complete secondary or higher	14,270	3.7	695	4.9	0.49	(0.44–0.54)	0.66	(0.60–0.74)
Household wealth quintile								
Poorest (reference)	91,614	22.6	8,115	9.2	1.00		1.00	
2nd	81,337	21.2	7,318	9.2	1.00	(0.96–1.04)	1.06	(1.02–1.11)
3rd	78,105	20.5	6,759	8.7	0.94	(0.90–0.98)	1.05	(1.00–1.09)
4th	73,501	19.3	5,984	8.2	0.88	(0.84–0.92)	1.07	(1.02–1.12)
Richest	69,377	16.4	4,571	6.5	0.69	(0.65–0.72)	1.00	(0.95–1.07)
Area of residence								
Urban	110,467	25.4	7,772	6.9	0.74	(0.72–0.77)	0.96	(0.92–1.00)
Rural (reference)	283,467	74.6	24,975	9.0	1.00		1.00	
Received skilled antenatal care								
No	130,102	34.0	17,645	13.7	2.60	(2.53–2.67)	2.11	(2.05–2.17)
Yes (reference)	263,832	66.0	15,102	5.8	1.00		1.00	
Skilled attendant at birth								
No	202,708	52.9	19,842	10.0	1.53	(1.48–1.58)	1.09	(1.05–1.13)
Yes (reference)	191,226	47.1	12,905	6.8	1.00		1.00	

**Fig. 3:**
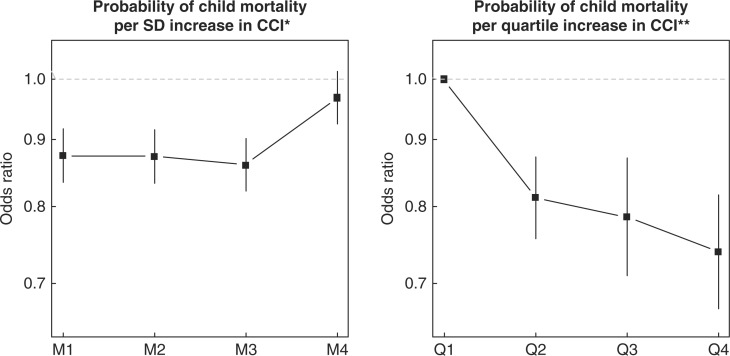
Odds ratios (OR) for the association of CCI with child mortality in 35 sub-Saharan African countries; OR per quartile increase in composite coverage index (CCI) (left) and per SD increase in CCI from 4 separate models (right). *Model 1 (M1) included country and survey period fixed effects; M2 added log pcGDP to M1; M3 added maternal- and child-level covariates to M2; M4 added indicators for whether mother received skilled antenatal care during pregnancy and presence of skilled attendant at birth. **Model includes country and survey period fixed effects, log pcGDP, and maternal- and child-level covariates; quartiles of CCI defined at the country level.

Table 3 shows the sample sizes, unadjusted and adjusted risks of child mortality by covariates for the child-level analyses. In these analyses, CCI was also associated with a reduction in mortality, with an odds ratio of 0.87 (95% CI: 0.84, 0.92) indicating a protective effect against under-5 mortality independent of survey period effects (Model 1 of Fig. 3a). The inclusion of log pcGDP to this model did not alter the effect size. In a third model that included all child and maternal covariates in addition to log pcGDP, CCI remained robustly associated with a reduction in child mortality (odds ratio: 0.86, 95% CI: 0.82–0.90). However, the effect became attenuated when individual-level indicators were included for whether the mother received skilled antenatal care during pregnancy and had the presence of a skilled attendant at birth (odds ratio: 0.97, 95% CI: 0.92–1.01, Model 4 of Fig. 3a). Without considering the individual-level indicators of intervention utilization, there was a graded and inverse association between CCI at the country level and probability of child mortality. Children from countries in the highest quartile of CCI coverage had the lowest probability of mortality conditional on all covariates (odds ratio 0.74, 95% CI: 0.67–0.82) (Fig. 3b).

**Table 3:** Coefficients of two ecological models predicting U5MR across 81 survey periods in 35 sub-Saharan African countries, 1992–2012

	Model 1		Model 2	
	
Variable	β	SE	β	SE
Survey period (ref = baseline, 0)				
1st repeated survey	−6.7	5.8	−7.1	5.9
2nd repeated survey	−36.3	7.4	−37.4	7.9
3rd repeated survey	−57.8	14.1	−59.6	15.0
% Composite coverage index (per SD increase)	−28.5	6.9	−29.0	7.1
Log GDP per capita (per SD increase)			5.2	12.8
Constant	144.7		145.2	

## Discussion

In this study, we explored the contribution of coverage of MNCH interventions to the declines in U5MR across 35 sub-Saharan African countries from 1990 to 2012. Improvements in MNCH coverage and interventions were strongly associated with reductions in child mortality; this association was universally consistent across the two types of data structures analyzed and regardless of the statistical specification. Analyses at the individual level, however, demonstrated that the CCI may only be a proxy for maternal/child-level utilization of health interventions as the protective effect of national-level CCI coverage attenuated after controlling for indicators of ANC and SBA.

This study has several limitations. First, given that DHS surveys are typically conducted only at intervals of 3–6 years (38), we were only able to study large changes in U5MR, and in some countries repeated surveys were not available, prohibiting a full time-series cross-sectional analysis. Further, some baseline surveys were conducted at different time periods especially if countries were only involved later in the DHS program and thus have had only one survey conducted. We decided to retain these countries in the levels analyses in order to make the full use of available data although our primary focus was on change in CCI and change in U5MR over time and such countries did not contribute to the change analyses. Finally, all of our models accounted for a survey-year variable to adjust for the different periods in which the surveys were conducted. Second, due to sample size restrictions in the ecological analyses, we could not model each indicator of MCNH interventions separately in a multivariable model, and instead we chose the CCI which is a composite index of eight different key interventions. Other potentially relevant determinants of U5MR were not examined in this study.

Third, we analyzed U5MR over the 3-year period preceding each survey. This method provided a balance between increasing precision in the estimates of U5MR but also allowing for some information on recent trends in U5MR to be revealed by shortening the traditional 5-year reference period (48). Fourth, the coverage of maternal, newborn, and child health interventions were also calculated from each of the surveys, using the 3-year reference period before each survey and coverage of interventions are self-reported by survey respondents. There are some limitations to the validity of self-reports of uptake of these interventions. Some studies have described acceptable validity of maternal reports for peripartum interventions in DHS/MICS surveys (49). Further, DHS uses a combination of maternal reports with other documentation, for example, the use of health cards to gather information on vaccination uptake. Despite these limitations, DHS and other household surveys have generally been found to have reasonable and perhaps better validity than officially reported data by service providers (50). A related limitation is that there remains some difficulty in establishing the timing of exposures and outcomes, as both were measured contemporaneously in the same survey. Further, although we chose a logistic regression analysis for the child-level models, a hazard model would have been another alternative. Regardless of model choice, there would be no additional information gained from the independent variables, given that the indicators were calculated using a 3-year window.

Fifth, the analyses did not include indicators of the incidence (or prevalence) of childhood diseases. Given the method in which the prevalence of diseases is captured in DHS (i.e. any diarrhea within 2 weeks preceding the survey), we were not confident that these would be comparable across countries, especially since surveys may have been conducted at different times and in different seasons. Finally, a more general limitation is that this study was based on estimates of U5MR. Any estimate of U5MR from survey data is subjected to sampling errors and will always be inferior to complete vital registration data (4). Countries where U5MR remains high and/or rates of mortality decline are slow typically lack comprehensive vital registration systems (51). Strengthening such systems is likely to improve future assessments of factors associated with declines in U5MR in sub-Saharan African countries.

The results presented in this study indicate a secular decline in U5MR in a majority of countries in sub-Saharan Africa over the past two decades. A large part of this decline can be explained by coverage of selected maternal, newborn, and child health interventions. On average, the increases in CCI correlated with decreases in U5MR; however, all countries did not fit this trend. For example, in Zimbabwe, Nigeria and Zambia, the CCI decreased between the baseline and repeated survey and in Zambia the U5MR decreased from 195.4 to 117.1 even though the CCI decreased from 67.4 to 61.8%. These findings suggest that other factors not considered here may also be influencing change in U5MR. Further, the CCI is a composite measure, and a decline in CCI may reflect that one of the components decreased over time while other components may have increased. We were not able to assess the association of each component of the CCI with U5MR, but it is likely that some components are more strongly associated than others. For example, our analysis presented in Fig. 3 suggests that antenatal care is particularly important in reducing U5MR. It is therefore possible that increases in coverage of some interventions but not other may result in an improvement in U5MR without a corresponding improvement in CCI. Other social improvements, such as improved access to clean drinking water and sanitation facilities, may also have an important role (29). Our analyses did not fully account for the variation in U5MR or child-level mortality, suggesting that other factors related to health systems as well as economic, social, or political factors play a role in influencing U5MR in sub-Saharan Africa.

It has been suggested that effective implementation of available, cost-effective MNCH interventions can prevent much of the current burden of under-5 mortality in low-income settings (52). However, many countries in sub-Saharan Africa are not on track to reach MDG-4 (7), which is likely related in part to the low levels of coverage of key interventions in the 1990s in many countries (37, 53). In the 2000s, global health initiatives and resources for health increased, and along with such increases came improvements in coverage of life-saving child health interventions in several countries (18, 41). We would therefore expect that progress toward MDG-4 in such settings, while lagging behind other areas, might likely continue into 2015 and beyond (4, 7).

It appears that health system improvements, including scaling up of key MNCH interventions, are a key explanation for reductions in U5MR in sub-Saharan Africa. For example, in Tanzania between 1999 and 2004–05, the coverage of interventions relevant to child survival improved substantially (54). In particular, vitamin A supplementation increased from 14% in 1999 to 85% in 2005, and other improvements also were seen: children sleeping under insecticide-treated nets increased from 10 to 29%, ORT for children increased from 57 to 70%, and exclusive breastfeeding for those younger than age 2 months increased from 58 to 70% (54).

Over this same period, Tanzania's national wealth (in GDP per person) increased by 93 international dollars, from $819 to $912 per person (or US$256–US$303). Improvements in the proportion of households living below the poverty line, in educational attainment, and in literacy rates improved only marginally during this time. Therefore, it is unlikely that growth in national wealth would account for much of the reduction in mortality, especially since poverty rates in Tanzania and other sub-Saharan African countries did not reduce dramatically over the study period.

Based on our child-level analyses, it appears that the coverage of health interventions have played a relatively more important role in reducing child mortality compared with the role of economic growth. However, it is not clear whether these improvements are being driving by supply side increases in the national or regional availability and coverage of health services and interventions or through increased demand and access at an individual level. Our inclusion of individual-level analogues of two components of CCI (ANC and SBA) was sufficient to attenuate the effect of CCI, suggesting that individual-level demand and access to interventions maybe the pathway where the improvements to child health can be gained. It also suggests that the projected gains in child mortality reductions from scaling up of coverage of the various interventions may be overstated unless these increases in coverage can be appropriately translated to individual-level utilization (55).

Although recent gains have been made in reducing under-5 mortality in sub-Saharan Africa, U5MR in this region continues to be the highest globally. While sub-Saharan Africa as a whole has reduced U5MR by 30%, this is less than half of the MDG-4 target. As the global health community considers both the strong likelihood that the MDG-4 targets are not going to be accomplished by 2015 (56, 57), and looks ahead to the post-MDG era (58), it is important to sustain efforts to reduce child mortality. For sub-Saharan Africa, a continued focus on fertility declines, improved health coverage, and greater equity in the coverage of proven life-saving interventions might be the key to reducing mortality.
